# Supplementary material for: BCL11A interacts with SOX2 to control the expression of epigenetic regulators in lung squamous carcinoma
Source: Nat Commun. 2018 Aug 20;9:3327. doi: 10.1038/s41467-018-05790-5 (PMC6102279; doi:10.1038/s41467-018-05790-5)
Supplement: Supplementary file 1 — Supplementary Information [file 41467_2018_5790_MOESM1_ESM.pdf]

BCL11A interacts with SOX2 to control the expression of epigenetic regulators in lung squamous carcinoma

Kyren A. Lazarus[1,7], Fazal Hadi[1,7], Elisabetta Zambon[1,7], Karsten Bach[1,7], Maria-Francesca Santolla[1,3], Julie K. Watson[6], Lucia L. Correia[2], Madhumita Das[8], Rosemary Ugur[1,7], Sara Pensa[1,7], Lukas Becker[1], Lia S. Campos, Graham Ladds[1], Pentao Liu[4], Gerard Evan[2], Frank McCaughan[2], John Le Quesne[8,9,10], Joo-Hyeon Lee[6], Dinis Calado[5] and Walid T. Khaled[1,7]

1. Department of Pharmacology, University of Cambridge, Cambridge, UK

2. Department of Biochemistry, University of Cambridge, Cambridge, UK

3. Department of Pharmacy, Health and Nutritional Sciences, University of Calabria, Rende, Italy

4. Wellcome Trust Sanger Institute, Cambridge, UK

5. The Francis Crick Institute, London, UK

6. WT-MRC Stem Cell Institute, University of Cambridge, UK

7. Cambridge Cancer Centre, Cambridge, UK

8. MRC Toxicology Unit, Lancaster Road, Leicester

9. Cancer Research Centre, University of Leicester

10. University Hospitals Leicester NHS trust

Correspondence to: Walid T. Khaled (wtk22@cam.ac.uk)

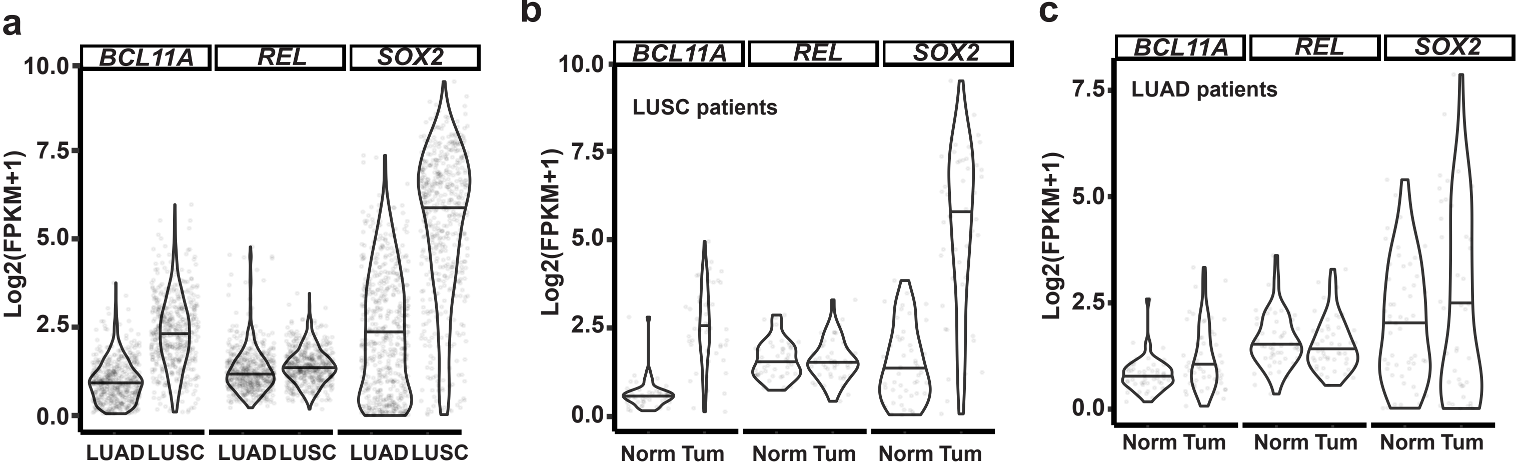

Supplementary Figure 1. BCL11A, SOX2, and REL expression in Lung TCGA dataset
(a) Violin plot showing BCL11A, SOX2 and REL expression in LUSC vs LUAD. FPKM, fragments per kilobase per million mapped reads. (b) Violin plot showing BCL11A, SOX2 and REL expression in LUSC tumour vs normal matched patients. (c) Violin plot showing BCL11A, SOX2 and REL expression in LUAD tumour vs normal matched patients.

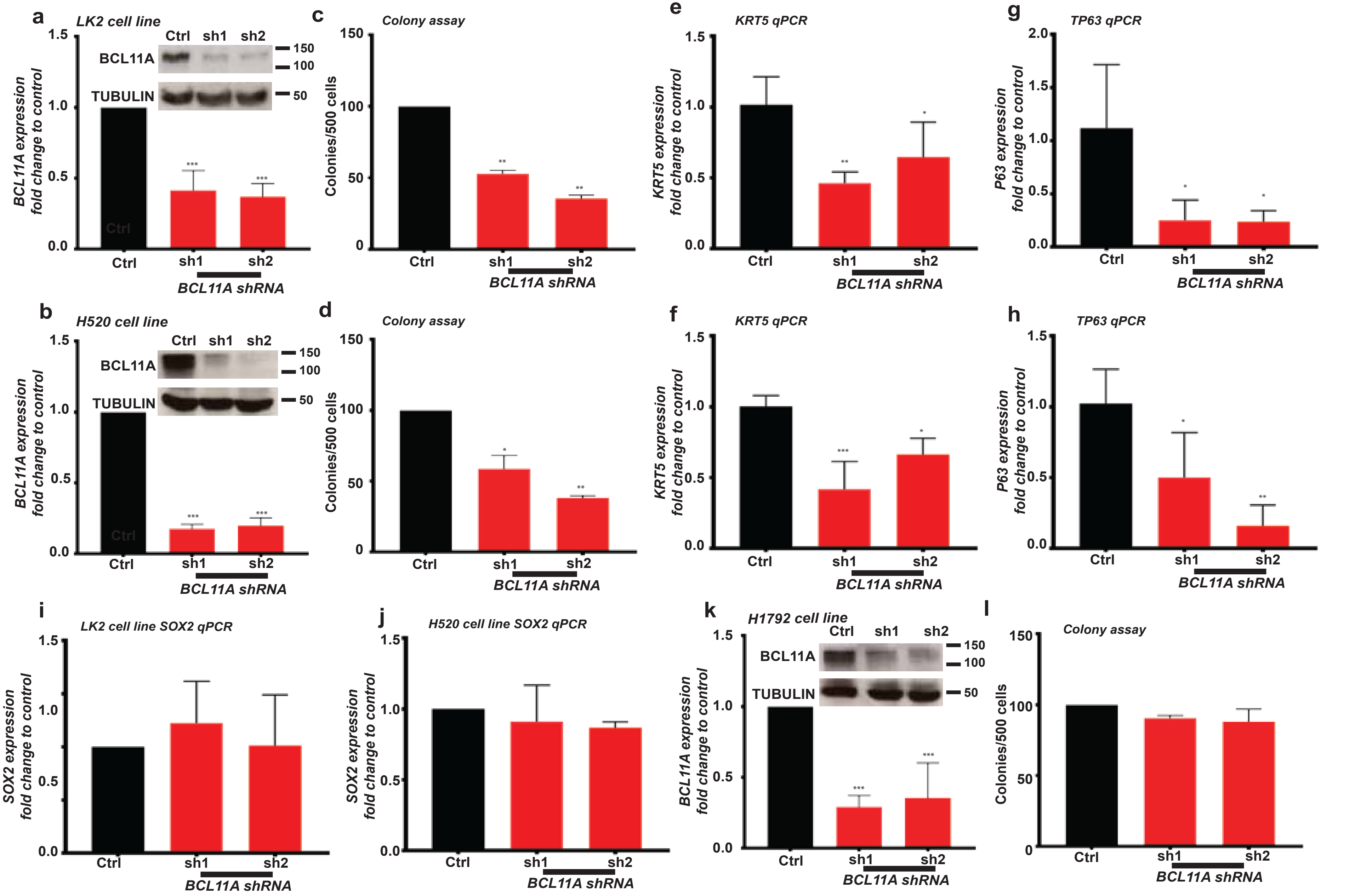

Supplementary Figure 2. BCL11A KD reduces squamous markers in LUSC cells
*(a and b) qPCR and western blot shows BCL11A reduction in LK2 (a) and H520 (b) cells transfected with shRNA1 and shRNA2 vectors. (c and d) Comparison of colony numbers in 3D matrigel assay from control, shRNA1 or shRNA2 in LK2 (c) and (d) H520 cells. Data presented as mean ± s.d. (n=3). (e and f) KRT5 expression is reduced in LK2 (e) and H520 (f) BCL11A-KD cells. (g and h) TP63 expression is reduced in LK2 (g) and H520 (h) BCL11A-KD cells. (i and j) SOX2 expression is unchanged in LK2 (i) and H520 (j) BCL11A-KD cells. (k) qPCR and western blot shows BCL11A reduction in H1792 cells transfected with shRNA1 and shRNA2 vectors. (l) Comparison of colony numbers in 3D matrigel assay from control, shRNA1 or shRNA2 in H1792 cells. Data presented as mean ± s.d. One way ANOVA with post Dunnett test performed, * indicates p<0.05 and ** p<0.005 and *** indicates p<0.001.*

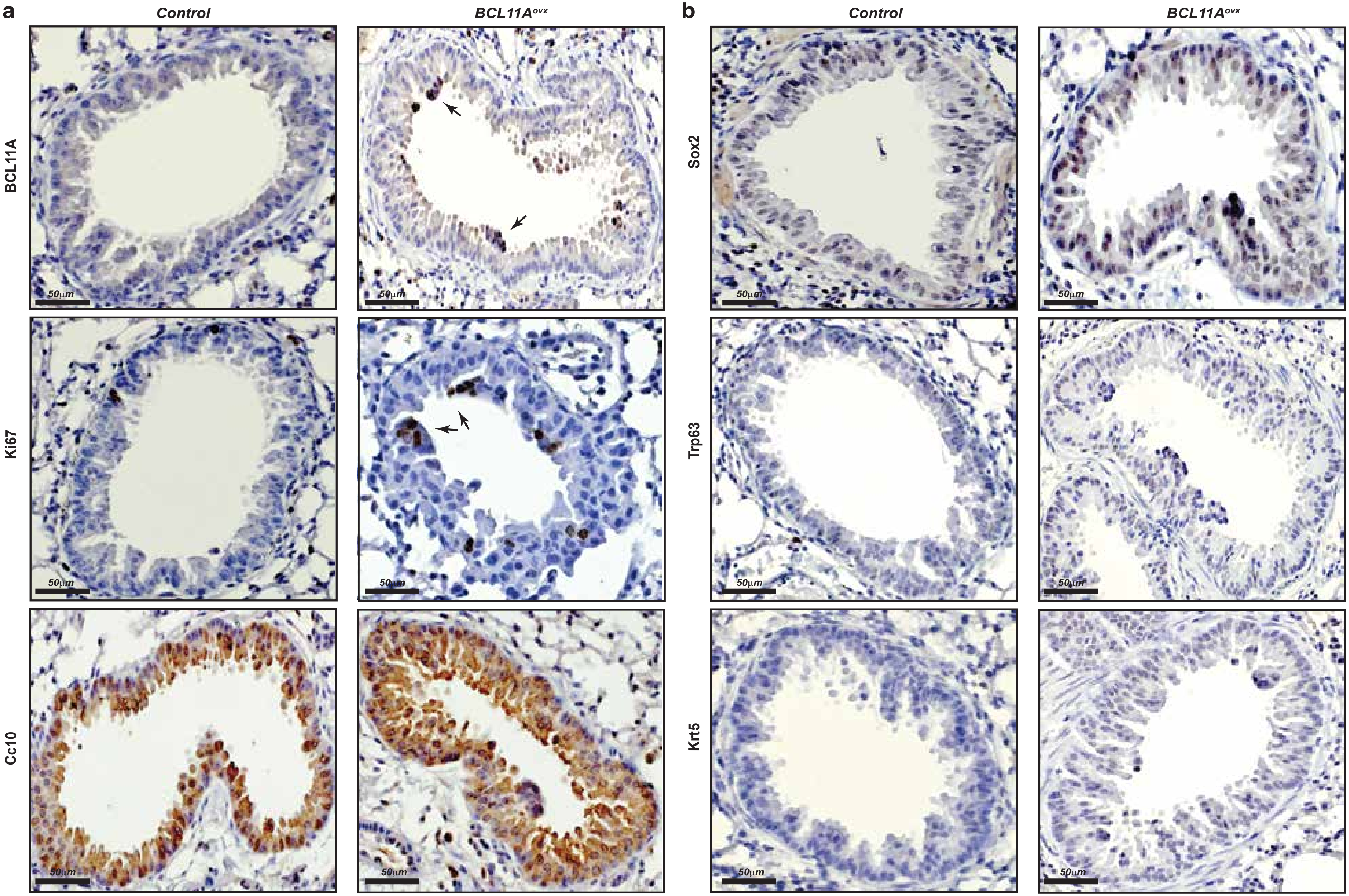

Supplementary Figure 3. Airways from the BCLL1Aovx mice demonstrate proliferative preneoplastic lesions
(a) Immunohistochemistry for BCL11A, Ki67 and Cc10 expression in control vs BCL11Aovx airways. BCL11A panel, arrows indicating positive staining especially in preneoplastic lesions. Ki67 panel, arrows indicating positive staining. (b) Sox2, Trp63 and Krt5 expression in control vs BCL11Aovx airways. Scale bar indicates 50 μm.

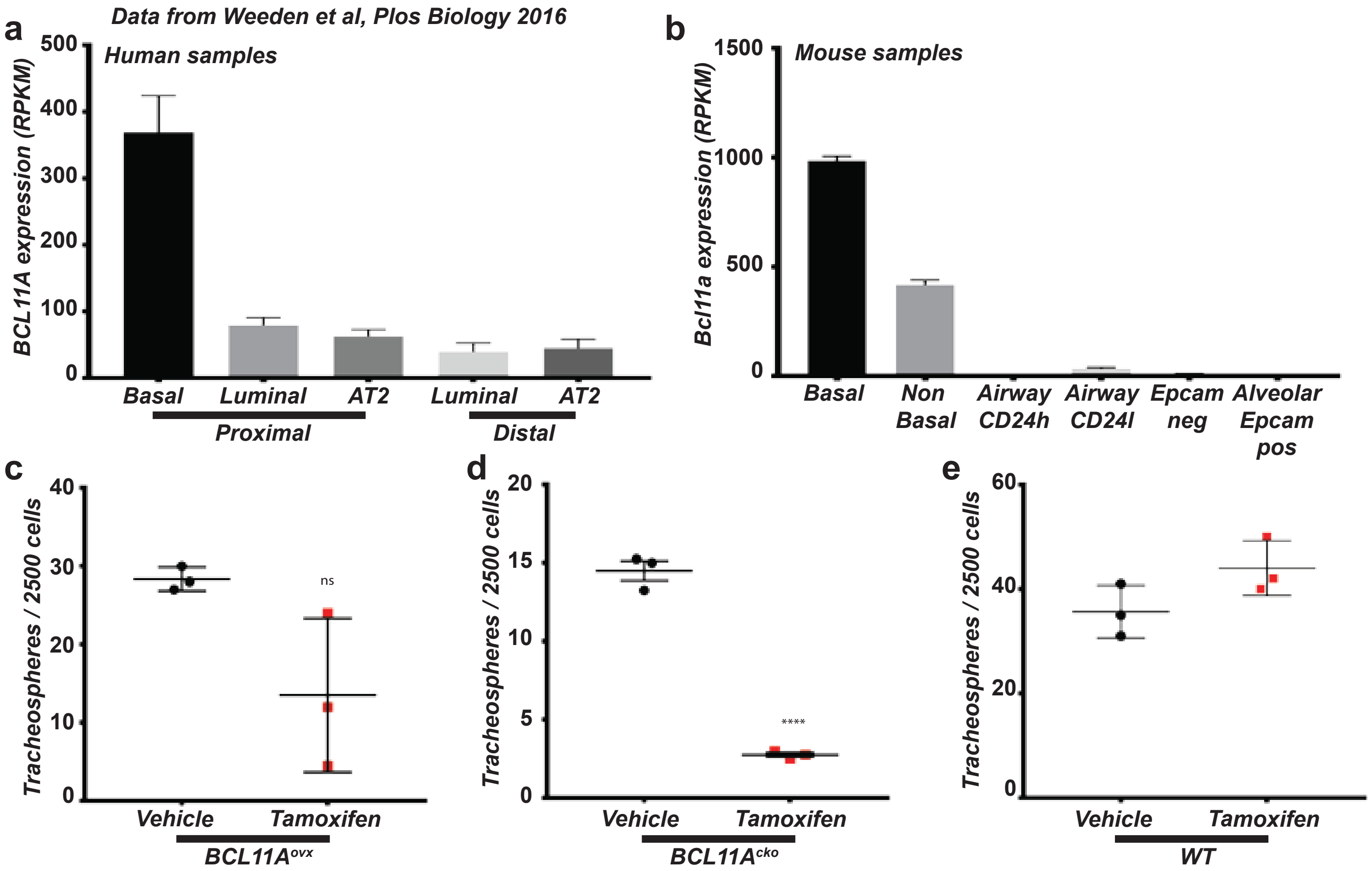

Supplementary Figure 4. Organoids from BCL11Aovx basal cells exhibit increase in squamous markers
(a) RNA-Seq data analysed from Weeden et al 22 indicating that in human samples FACS fraction labelled as proximal BCs has approximately 400 fold increase in human BCL11A levels in comparison to other epithelial fractions. (b) RNA-Seq data from the same dataset indicating that mouse Bcl11a is approximately 500-1000 fold higher in basal fractions compared to other epithelial subtypes. (c, d and e) Quantification of organoids in vehicle vs tamoxifen treated (c) BCL11Aovx, (d) BCL11Acko and (e) WT organoids. Data presented as mean ± s.d. Paired student t-test performed, * indicates $p<0.05$ and ** $p<0.005$ and *** indicates $p<0.001$.

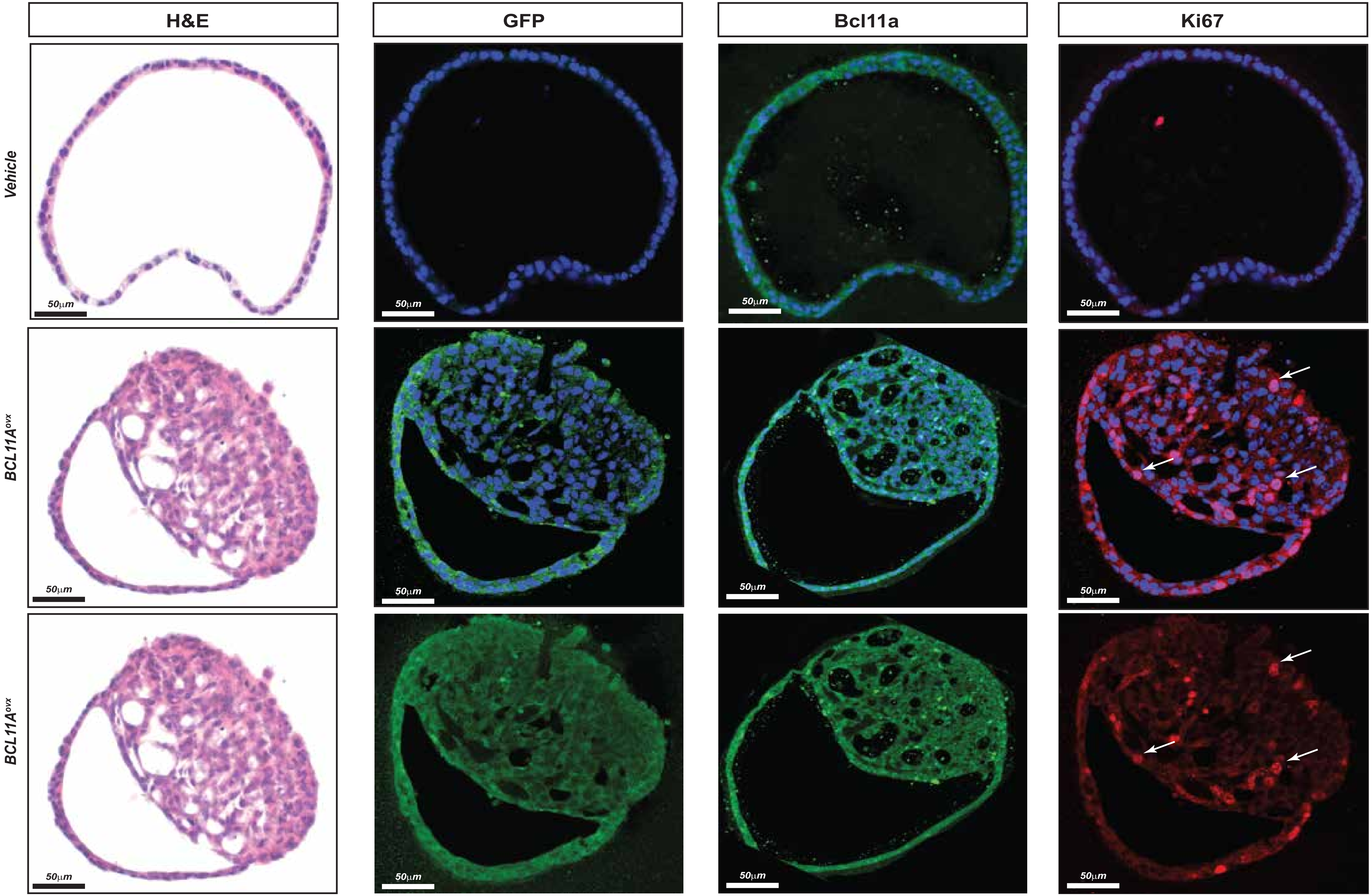

Supplementary Figure 5. Organoids from the BCL11Aovx basal cells display an abnormal proliferative phenotype
Vehicle and tamoxifen treated BCL11Aovx organoids stained with H&E, GFP (which is also expressed if the LSL is efficiently excised), Bcl11a and Ki67. Nuclei stained by DAPI illustrated in blue. Arrows indicate positive staining. Scale bar indicates 50 μm.

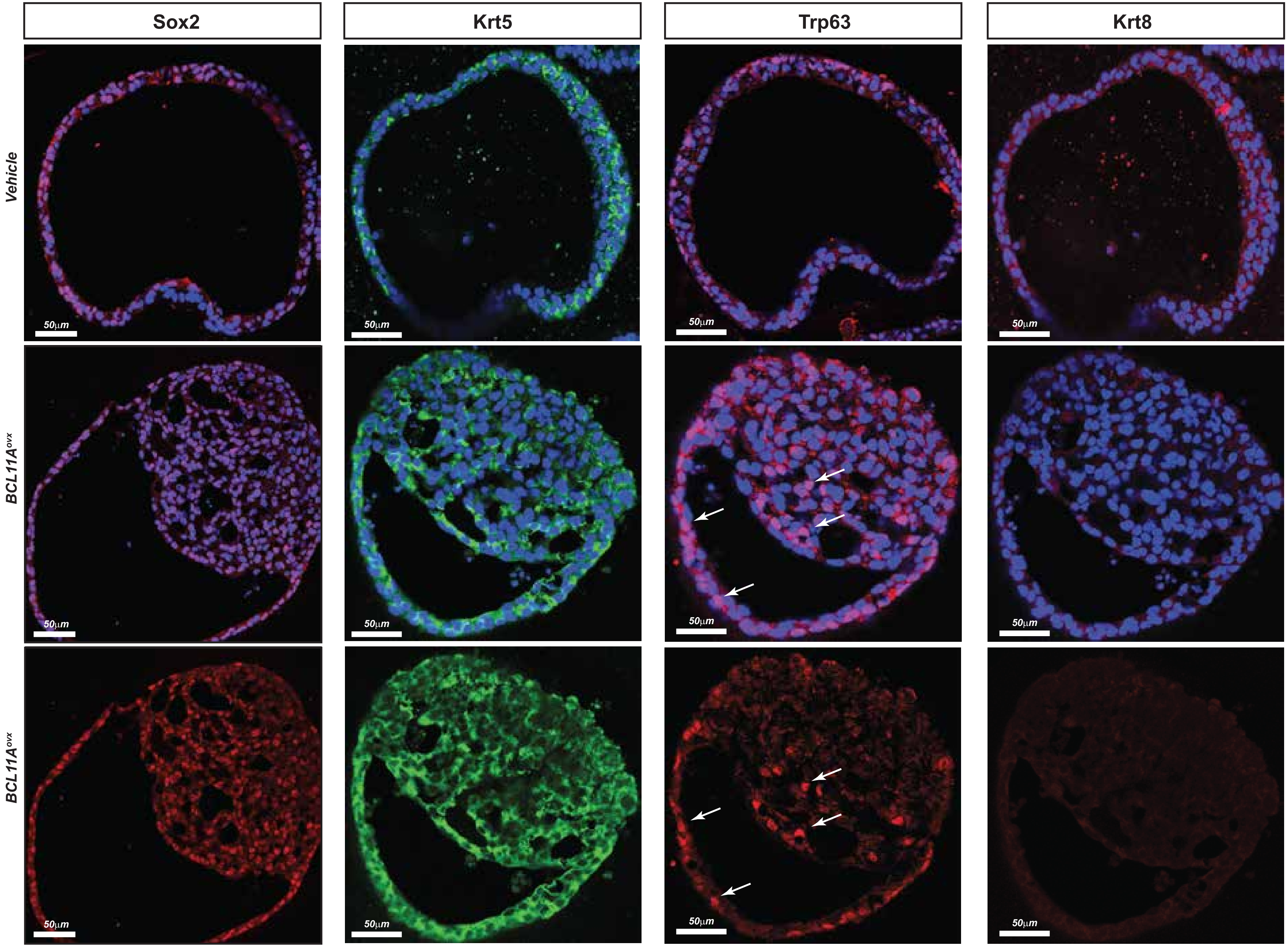

Supplementary Figure 6. BCL11A overexpression inhibits organoid luminal differentiation
Vehicle and tamoxifen treated BCL11Aovx organoids stained with Sox2, Krt5, Trp63 and Krt8. Nuclei stained by DAPI illustrated in blue. Arrows indicate positive staining. Scale bar indicates 50 µm.

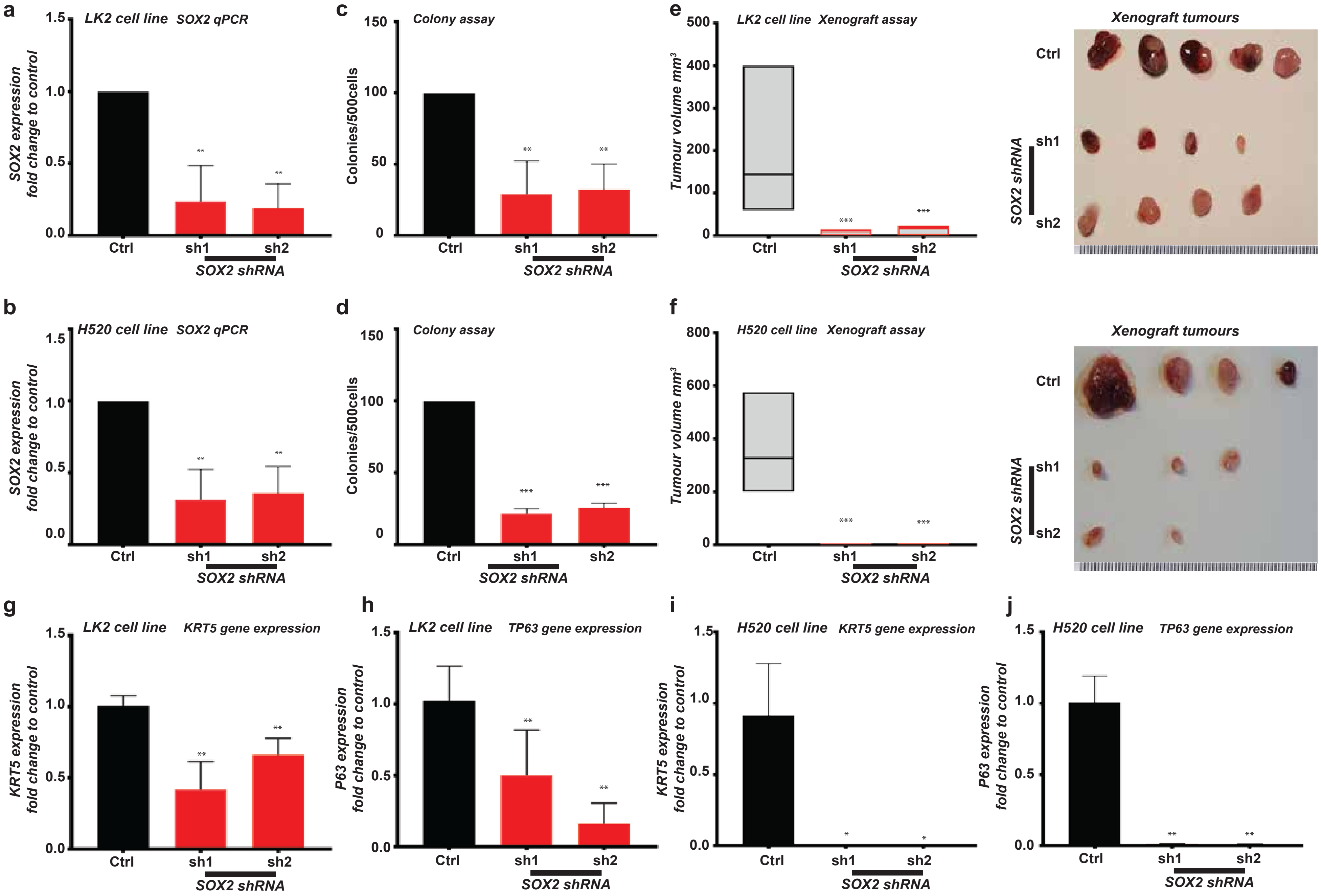

Supplementary Figure 7. SOX2 KD affects LUSC cell lines tumourigenicity
*(a and b) Graph showing SOX2 expression in SOX2-KD LK2 (a) and H520 (b) cells. (c and d) Graph showing decrease in colony numbers in SOX2-KD LK2 (c) and H520 (d) cells. (e and f) Graph depicting reduction in tumour size observed when SOX2 shRNA1 or shRNA2 transfected LK2 (e) and H520 (f) cells are injected subcutaneously into mice compared with control. Five (LK2) and four (H520) mice per cell line were monitored for 15 days after which tumours were removed and measured. On the right are images showing actual tumours measured. Data presented as mean ± s.d. One way ANNOVA with post Dunnett test performed, * indicates p<0.05 and ** p<0.005 and *** indicates p<0.001. (g and h) KRT5 expression is reduced in LK2 (g) and H520 (h) BCL11A-KD cells. (i and j) P63 expression is reduced in LK2 (i) and H520 (j) BCL11A-KD cells.*

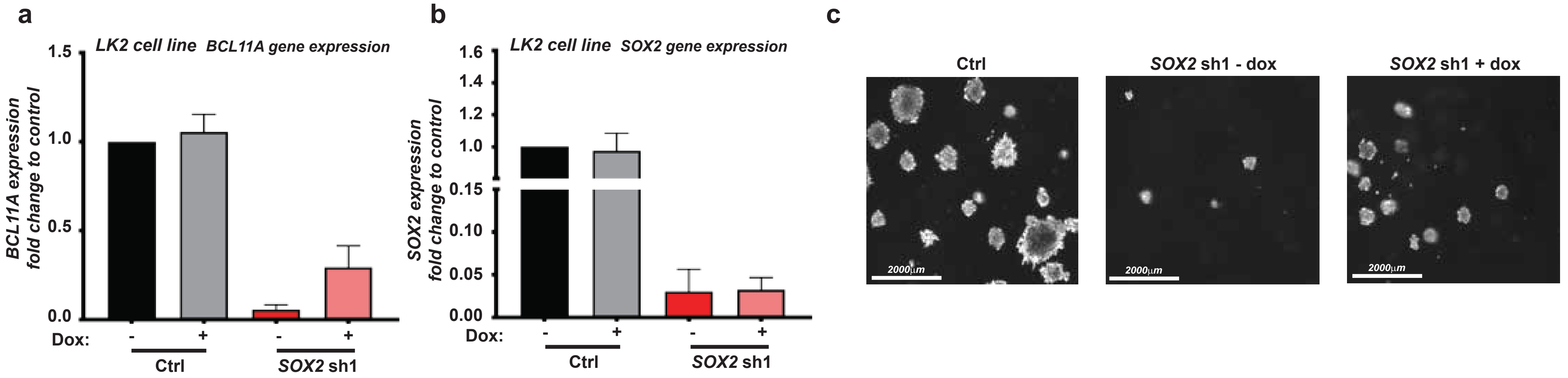

Supplementary Figure 8. BCL11A is required for SOX2 mediated LUSC phenotype
(a and b) Graph showing BCL11A (a) and SOX2 (b) expression in BCL11A rescue in SOX2-KD cells. Dox inducible BCL11A overexpression vector was transfected into control and SOX2 shRNA1 LK2 cells and Dox treatment was performed for 48 hours. (c) Images from 3D matrigel experiment showing reduction in colony numbers after SOX2-KD and partial rescue after BCL11A overexpression.

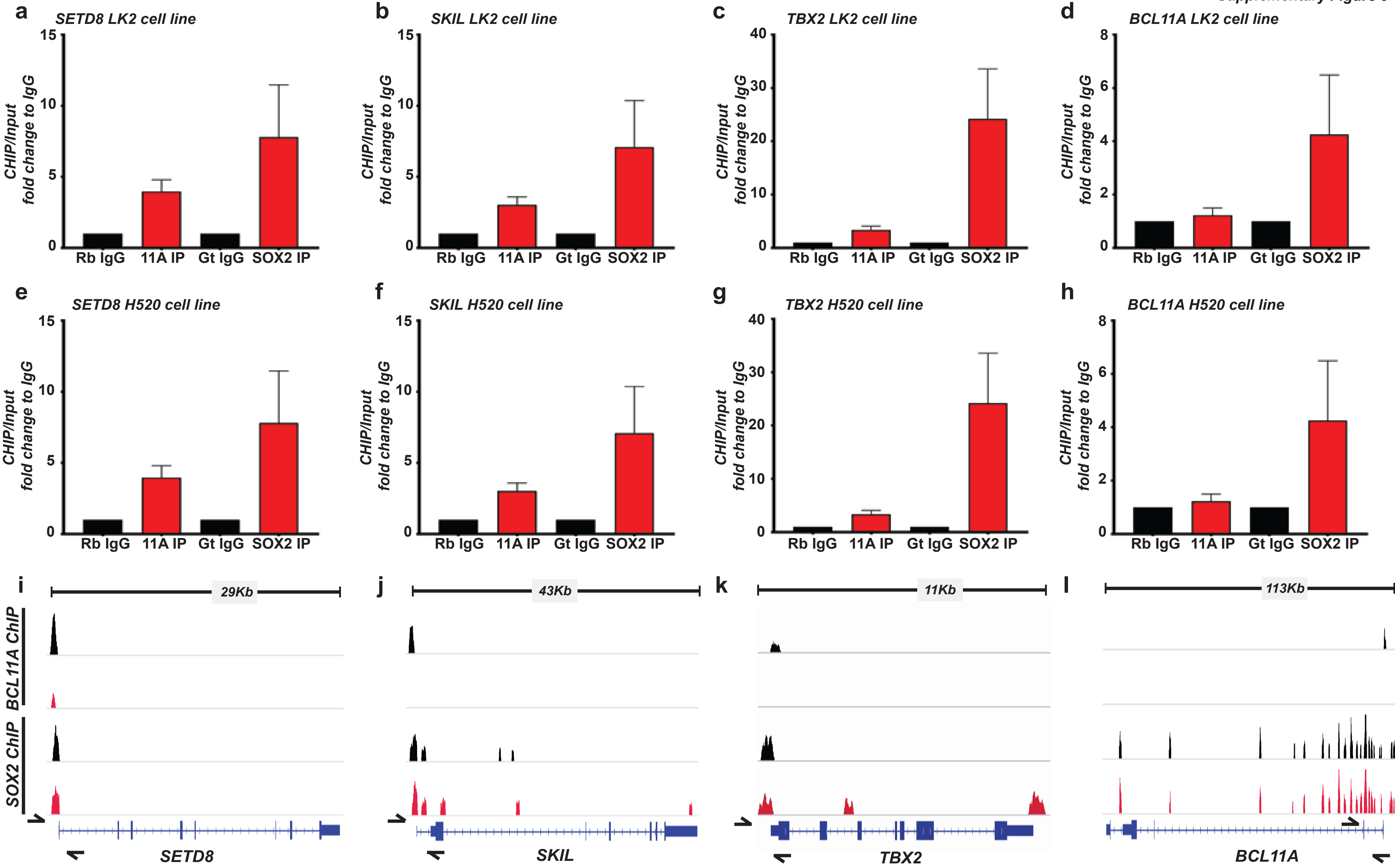

Supplementary Figure 9. BCL11A and SOX2 bind to common loci on the genome
(a-h) ChIP-qPCR showing enrichment for the indicated primers on SETD8, SKIL, TBX2 and BCL11A regions after Chip pull down using anti-BCL11A or anti-SOX2 antibodies in LK2 and H520 cell lines. (i-l) Schematic of amplicon locations for ChIP-qPCR validations performed in this study. Arrows represent location of primers used. Error bars represent mean ± s.e.m.; n = 3 (technical replicates).

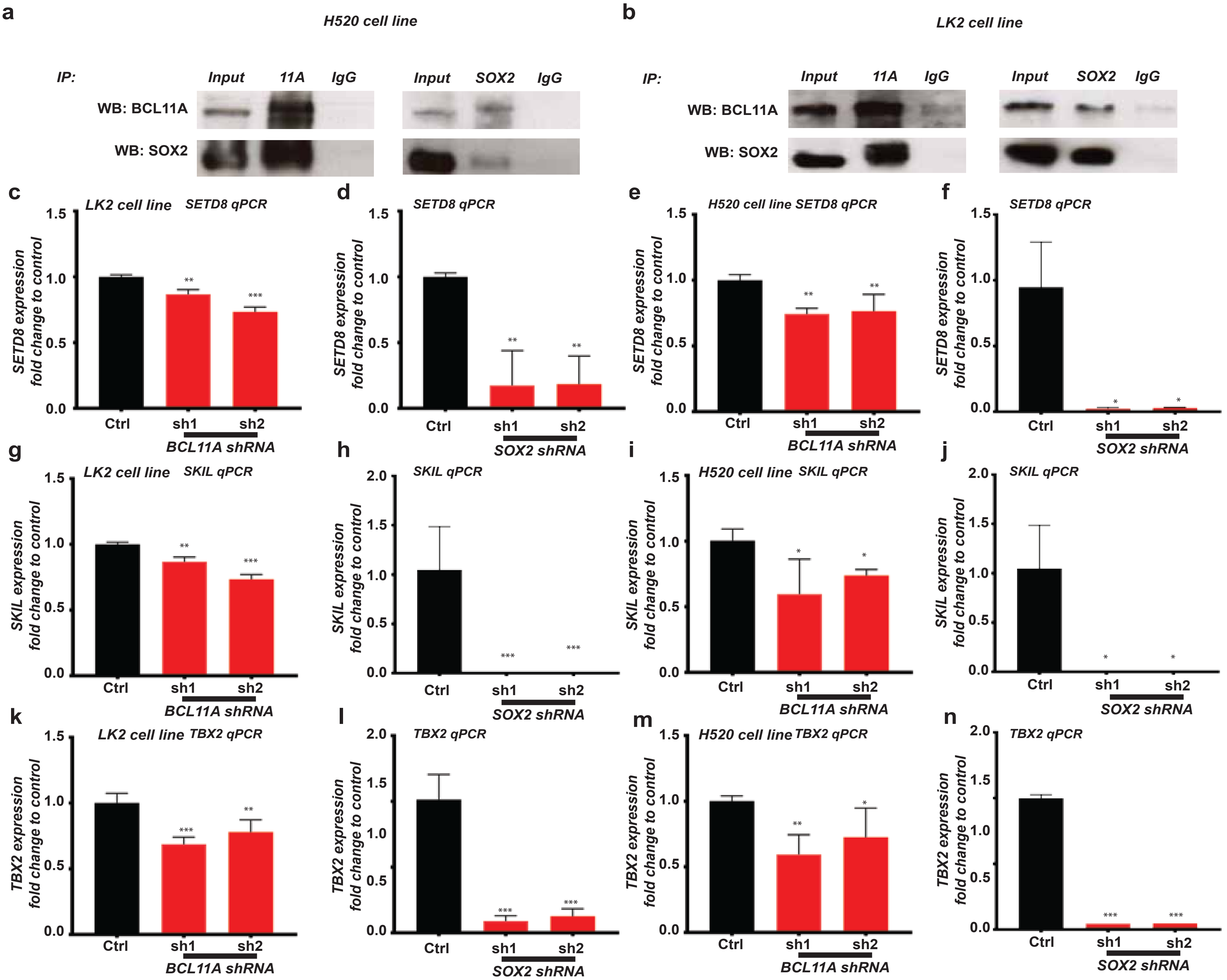

Supplementary Figure 10. BCL11A and SOX2 co-regulate transcriptional regulators
*(a and b) Co-Immunoprecipitation of endogenous BCL11A and SOX2 proteins in H520 (a) and LK2 (b) cell lines. (c-f) Graph depicting SETD8 gene expression in LK2, H520 BCL11A-KD and LK2, H520 SOX2 KD cells. (g-j) SKIL gene expression in BCL11A-KD or SOX-KD LK2 and H520 cell lines. (k-n) TBX2 gene expression in BCL11A-KD or SOX2-KD LK2 and H520 cell lines. Data presented as mean ± s.d. (n=3). One way ANOVA with post Dunnett test performed, * indicates p<0.05 and ** p<0.005 and *** indicates p<0.001.*

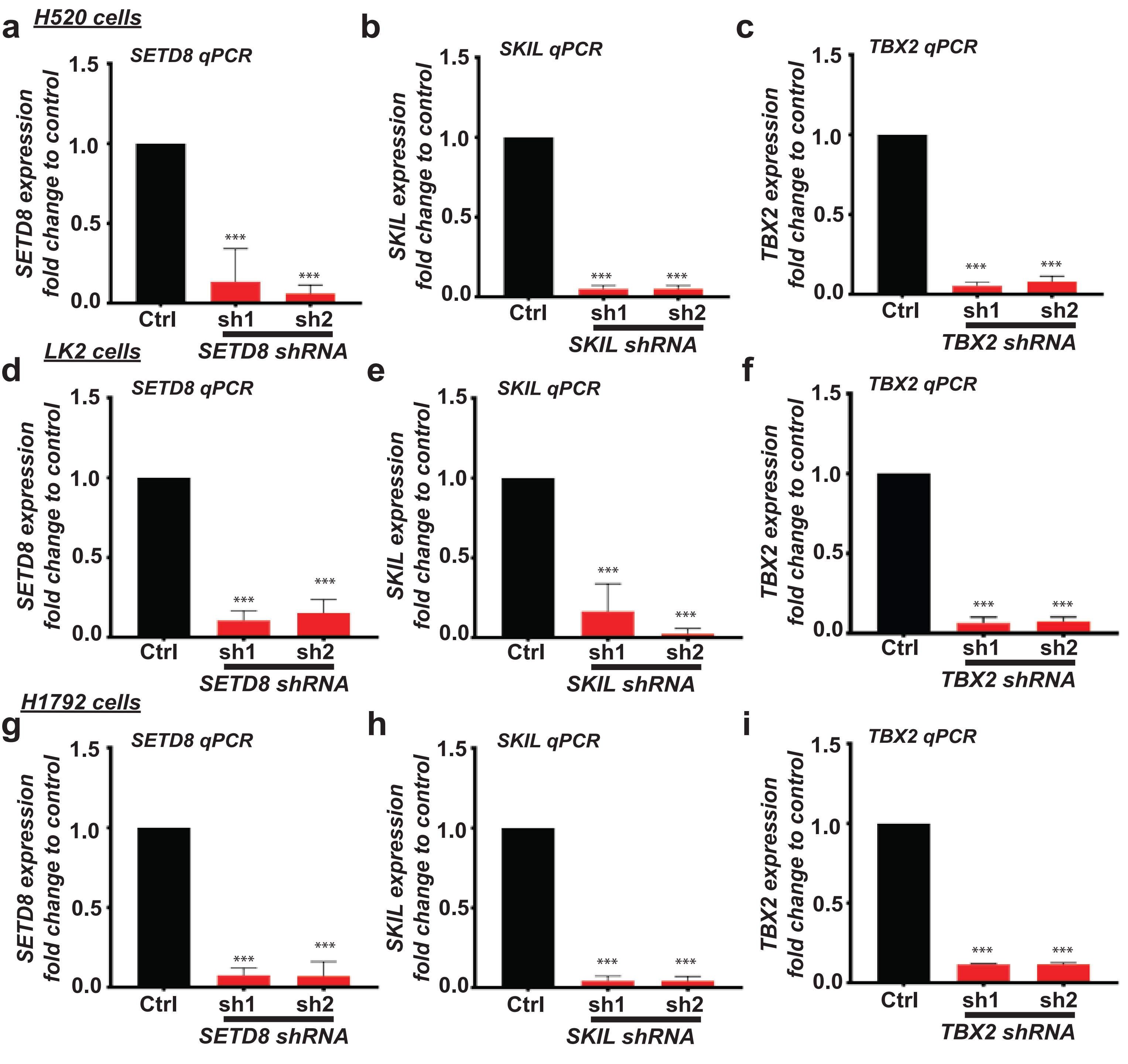

Supplementary Figure 11. Generation of SETD8, SKIL and TBX2 KD cells
(a-c) SETD8 gene expression in SETD8-KD (a) , SKIL gene expression in SKIL-KD (b), TBX2 gene expression in TBX2-KD (c) H520 cells.
(d-f) SETD8 gene expression in SETD8-KD (d) , SKIL gene expression in SKIL-KD (e), TBX2 gene expression in TBX2-KD (f) LK2 cells.
(g-i) SETD8 gene expression in SETD8-KD (g) , SKIL gene expression in SKIL-KD (h), TBX2 gene expression in TBX2-KD (i) H1792 cells.
*Data presented as mean ± s.d. (n=3). One way ANOVA with post Dunnett test performed, * indicates $p<0.05$ and ** $p<0.005$ and *** indicates $p<0.001$.*

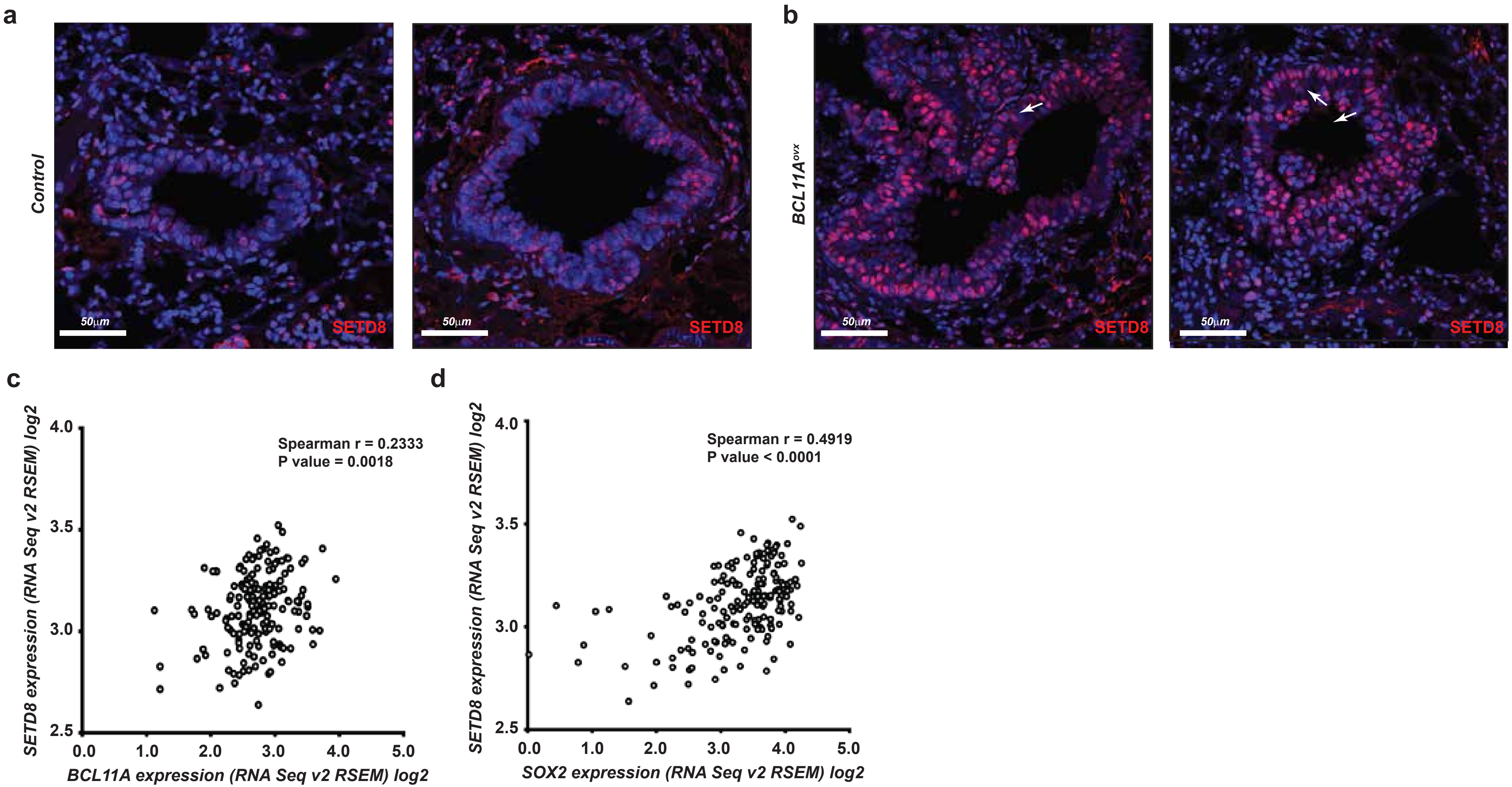

Supplementary Figure 12. SETD8 correlates with BCL11A and SOX2 in LUSC patients
(a and b) SETD8 immunofluorescence in control or BCL11AOVX mouse airways. Scale bar indicates 50 μm. (c) Scatter plot showing SETD8 and BCL11A expression in TCGA patient tumour samples. (d) Scatter plot showing SETD8 and SOX2 expression in TCGA patient tumour samples.

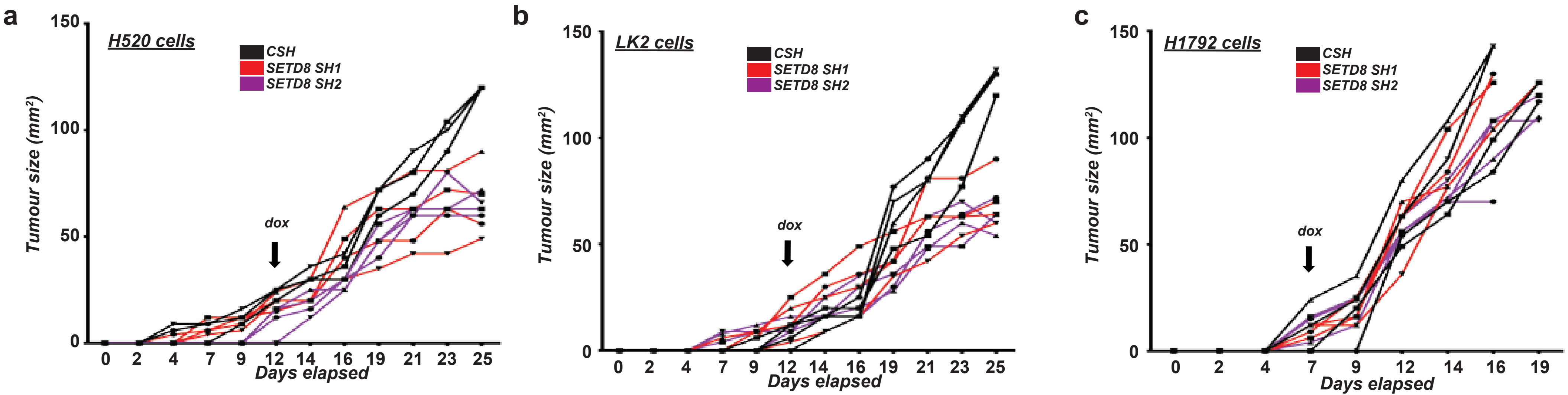

Supplementary Figure 13. SETD8-KD confers a survival disadvantage to xenograft tumours
Tumour kinetics of individual mice injected with either control or SETD8-KD vectors in H520 (a), LK2 (b) or H1792 (c) cells.

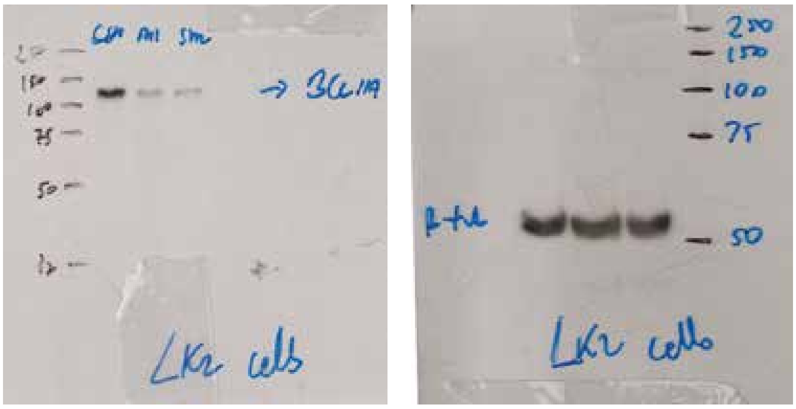

Western blot used for Supplementary Figure 2a

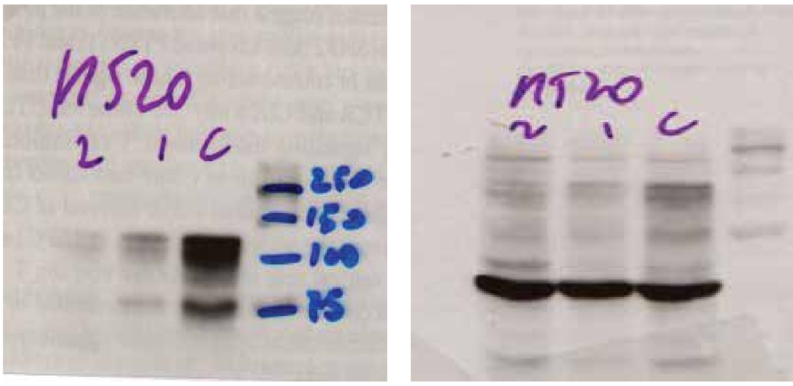

Western blot used for Supplementary Figure 2b

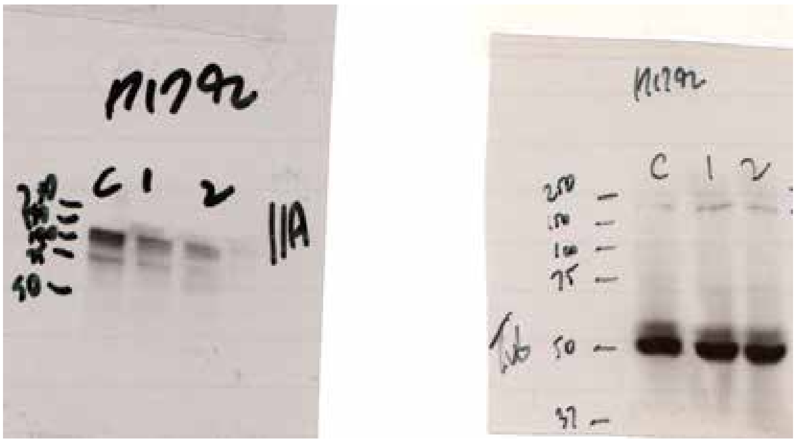

Western blot used for Supplementary Figure 2c

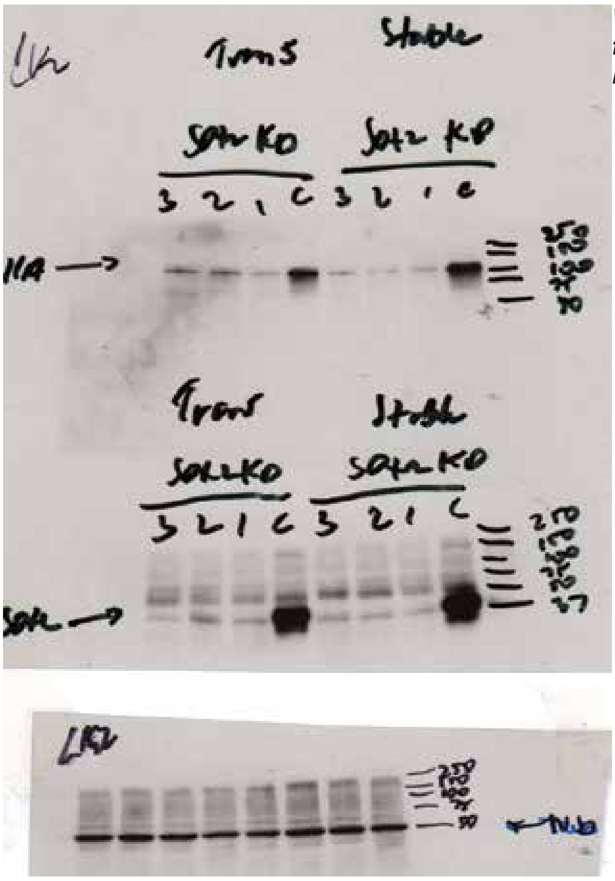

Western blot used for Supplementary Figure 3a

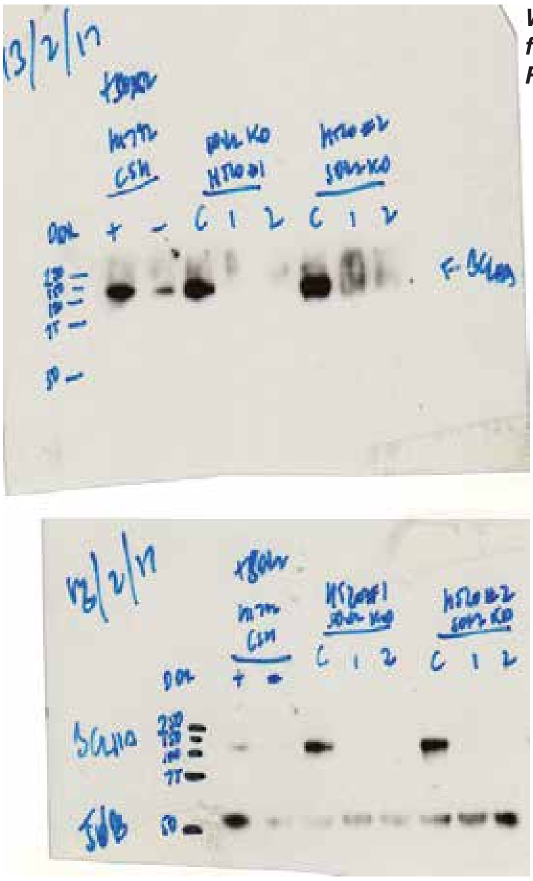

Western blot used for Supplementary Figure 3b

Supplementary Figure 14. Uncropped western blot images

Western blot used for Figure 3e

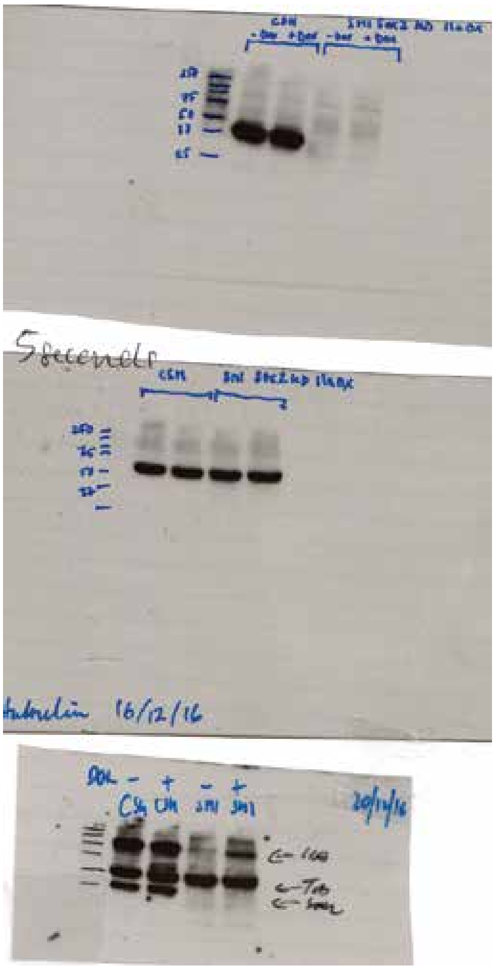

Western blot used for Supplementary Figure 10a

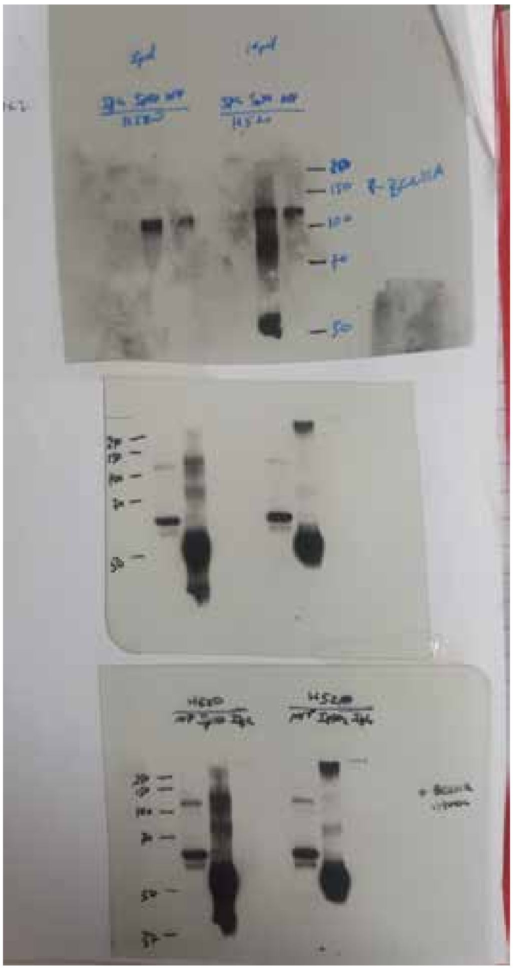

Western blot used for Supplementary Figure 10a

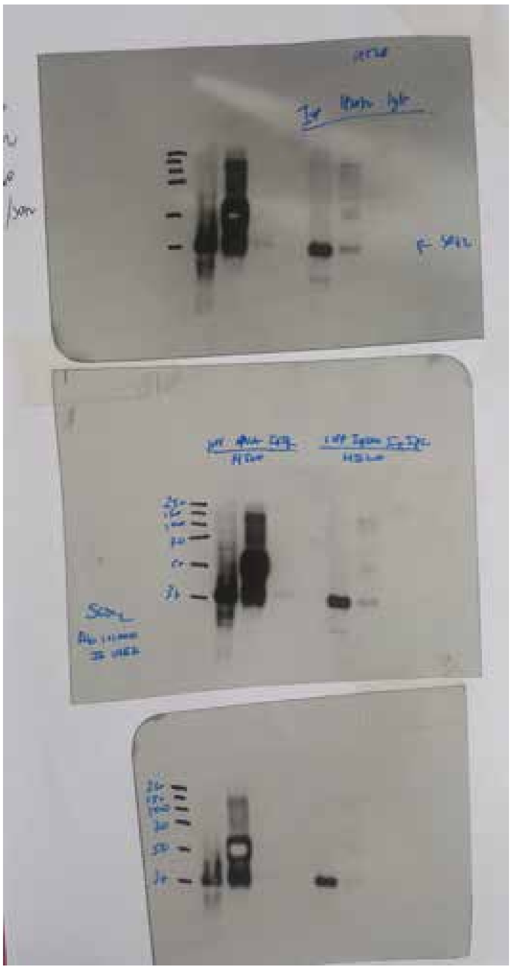

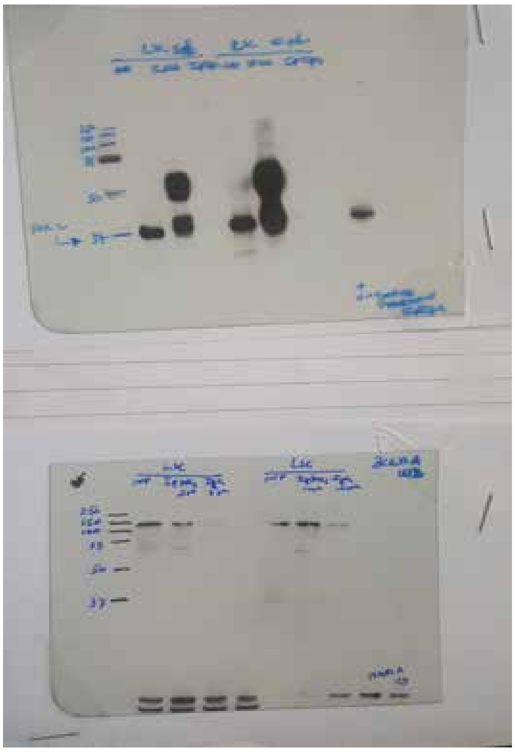

Western blot used for Supplementary Figure 10b

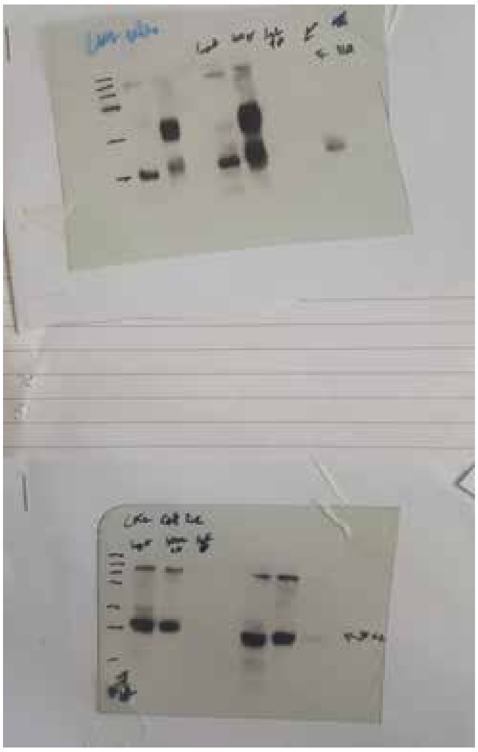

Western blot used for Supplementary Figure 10b

Supplementary Figure 15. Uncropped western blot images
