# Supplementary material for: BCL11A interacts with SOX2 to control the expression of epigenetic regulators in lung squamous carcinoma
Source: Nat Commun. 2018 Aug 20;9:3327. doi: 10.1038/s41467-018-05790-5 (PMC6102279; doi:10.1038/s41467-018-05790-5)
Supplement: Supplementary file 3 — Description of Additional Supplementary Files [file 41467_2018_5790_MOESM3_ESM.pdf]

Description of Additional Supplementary Files

Supplementary Data 1: Nearest downstream genes to BCL11A only peaks.

Supplementary Data 2: Nearest downstream genes to SOX2 only peaks.

Supplementary Data 3: Nearest downstream genes to overlap between BCL11A only and SOX2 only peaks.

Supplementary Data 4: List of primers used for genotyping.

Supplementary Data 5: List of qPCR primers used in this study.

Supplementary Data 6: List of CHIP qPCR primers used in this study.
